# Protein-Based Rechargeable and Replaceable Antimicrobial and Antifouling Coatings on Hydrophobic Food-Contact Surfaces

**DOI:** 10.1021/acsabm.3c01247

**Published:** 2024-02-28

**Authors:** Jiahan Zou, Jody Wong, Chih-Rong Lee, Nitin Nitin, Luxin Wang, Gang Sun

**Affiliations:** † Department of Biological and Agricultural Engineering, University of California, One Shields Avenue, Davis, California 95616, United States; ‡ Department of Food Science and Technology, University of California, One Shields Avenue, Davis, California 95616, United States

**Keywords:** *N*-halamine, fresh produce, foodborne pathogen, biofilm, gelatin, soy protein hydrolysate, tannic acid

## Abstract

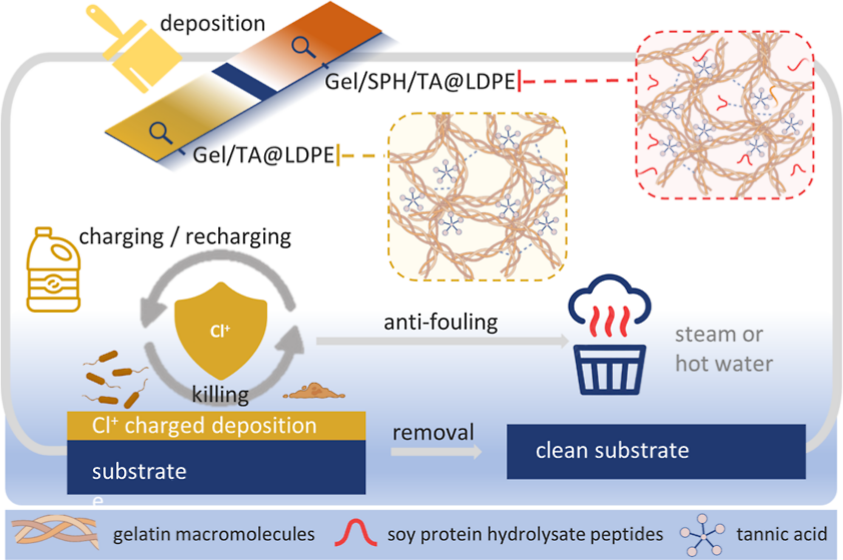

The growing concerns regarding foodborne illnesses related to fresh produce accentuate the necessity for innovative material solutions, particularly on surfaces that come into close contact with foods. This study introduces a sustainable, efficient, and removable antimicrobial and antifouling coating ideally suited for hydrophobic food-contact surfaces such as low-density polyethylene (LDPE). Developed through a crosslinking reaction involving tannic acid, gelatin, and soy protein hydrolysate, these coatings exhibit proper stability in aqueous washing solutions and effectively combat bacterial contamination and prevent biofilm formation. The unique surface architecture promotes the formation of halamine structures, enhancing antimicrobial efficacy with a rapid contact killing effect and reducing microbial contamination by up to 5 log_10_ cfu·cm^–2^ against both *Escherichia coli* (Gram-negative) and *Listeria innocua* (Gram-positive). Notably, the coatings are designed for at least five recharging cycles under mild conditions (pH6, 20 ppm free active chlorine) and can be easily removed with hot water or steam to refresh the depositions. This removal process not only conveniently aligns with existing sanitation protocols in the fresh produce industry but also facilitates the complete eradication of potential developed biofilms, outperforming uncoated LDPE coupons. Overall, these coatings represent sustainable, cost-effective, and practical advancements in food safety and are promising candidates for widespread adoption in food processing environments.

## 1 Introduction

The rising incidence of foodborne illnesses linked to fresh produce highlights an urgent need to address cross-contamination in postharvest processing and storage facilities. These facilities are potential hotspots of contamination and cross-contamination, given the diversity of food products processed daily.^1−5^ The risk of contamination or cross-contamination can be further enhanced by the formation of biofilms and the potential association of pathogens with biofilms, complex microbial communities that adhere to surfaces, thereby serving as potential reservoirs for persistent pathogens.^6−8^ In postharvest processing and storage facilities, surfaces made of stainless steel and plastic commonly come into direct contact with food. Importantly, hydrophobic surfaces such as low-density polyethylene (LDPE) in stackable containers, conveyor belts, and other equipment have been identified as particularly conducive to biofilm formation, necessitating enhanced antimicrobial measures.^9−11^

One promising strategy for reducing contamination risks involves the application of antimicrobial coatings to these food-contacting surfaces. Among various antimicrobial agents, *N*-halamines have gained attention for their capacity to generate active antimicrobial species upon interaction with microorganisms. Current *N*-halamine materials are effective in antimicrobial applications but are mostly made of synthetic substrates.^12−17^ A promising alternative approach is the use of biobased materials like proteins to form *N*-halamine structures, which can offer the desired functions and environmental benefits to food-contact surfaces.^18^ Proteins such as gelatin and soy protein hydrolysate (SPH), rich in primary and secondary amines, are attractive substrates for functionalization with *N*-halamines.^19−21^ Tannic acid (TA) serves as a food-grade crosslinking agent, stabilizing these proteins through Michael addition or Schiff base reactions.^22^ The chlorine-rechargeable properties of the proteins are crucial, allowing for prolonged antimicrobial activity and practicality in dynamic food processing environments.

In this paper, we propose a rechargeable and removable antimicrobial coating system based on TA-crosslinked gelatin and SPH. To promote effective adhesion of this bioactive layer to hydrophobic LDPE surfaces, an atmospheric plasma treatment is utilized.^23^ Gelatin serves as a matrix to form the skeletal structure of the coating and provides available sites for converting amino and peptide bonds to the corresponding halamine structures after chlorination with bleach. SPH, on the other hand, has a lower molecular weight and higher solubility, and uncrosslinked SPH acts as a temporary filler in the coating system, increasing the internal surface areas available for chlorination and the formation of halamine structures, leading to enhanced antimicrobial activity. Crosslinking of the protein molecules by TA results in a unique structural interplay, ensuring the coatings are robust in a low-temperature aqueous system but soluble and removable in a hot aqueous solution. We hypothesize that such coatings, made of gelatin, SPH, and TA, can serve as durable and effective barriers against microbial contamination, thereby significantly enhancing the safety and quality of the food supply chain.

## 2 Results and Discussion

### 2.1 Development of Protein-Based *N*-Halamine Deposition Systems

**Figure 1:**
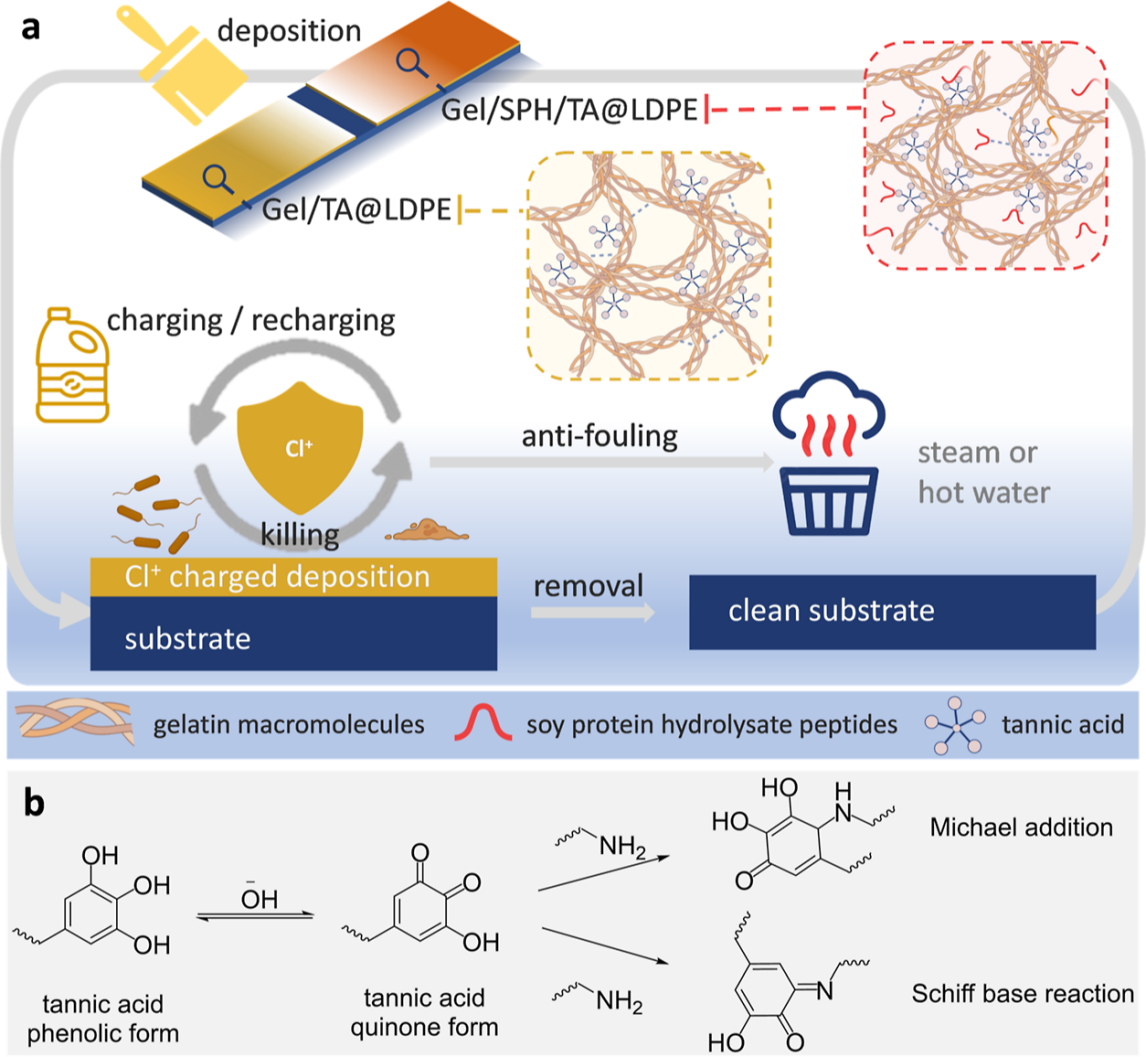
(a) Schematics of Gel/TA@LDPE and Gel/SPH/TA@LDPE systems in overall coating applications. (b) Crosslinking reaction mechanisms of TA with proteins.

Figure 1a illustrates the structures of the proposed antimicrobial protein coating systems and their typical deposition–application cycle. Within the cycle, LDPE is first atmospheric plasma treated, coated with the developed protein solution systems, and charged with active chlorine after drying. The charged coating releases active chlorine with antimicrobial functions. The deposition system can undergo multiple charge–release cycles with a bleach solution containing sufficient active chlorine content. Following a cycle’s completion, the formed coating layer can be effortlessly detached during normal cleaning processes using steam or a hot water rinse. Two coating systems derived from food ingredients were developed and evaluated: Gel/TA@LDPE and Gel/SPH/TA@LDPE, which are based on TA-crosslinked gelatin (Gel) and TA-crosslinked Gel/SPH composite networks, respectively. For the formulation of these coatings, TA was mixed with protein solutions at a pH of 8, initiating the oxidation of TA’s phenolic compounds into quinones and the subsequent crosslinking with proteins (Gel or Gel and SPH) through the Michael addition or Schiff base reactions, as illustrated in Figure 1b.^24^ The protein-based coatings can be readily functionalized to *N*-halamine structures by exposure to sodium hypochlorite or diluted household beach solutions, converting from Gel/TA@LDPE and Gel/SPH/TA@LDPE to Gel/TA-Cl^+^@LDPE and Gel/SPH/TA-Cl^+^@LDPE systems, respectively.

**Figure 2:**
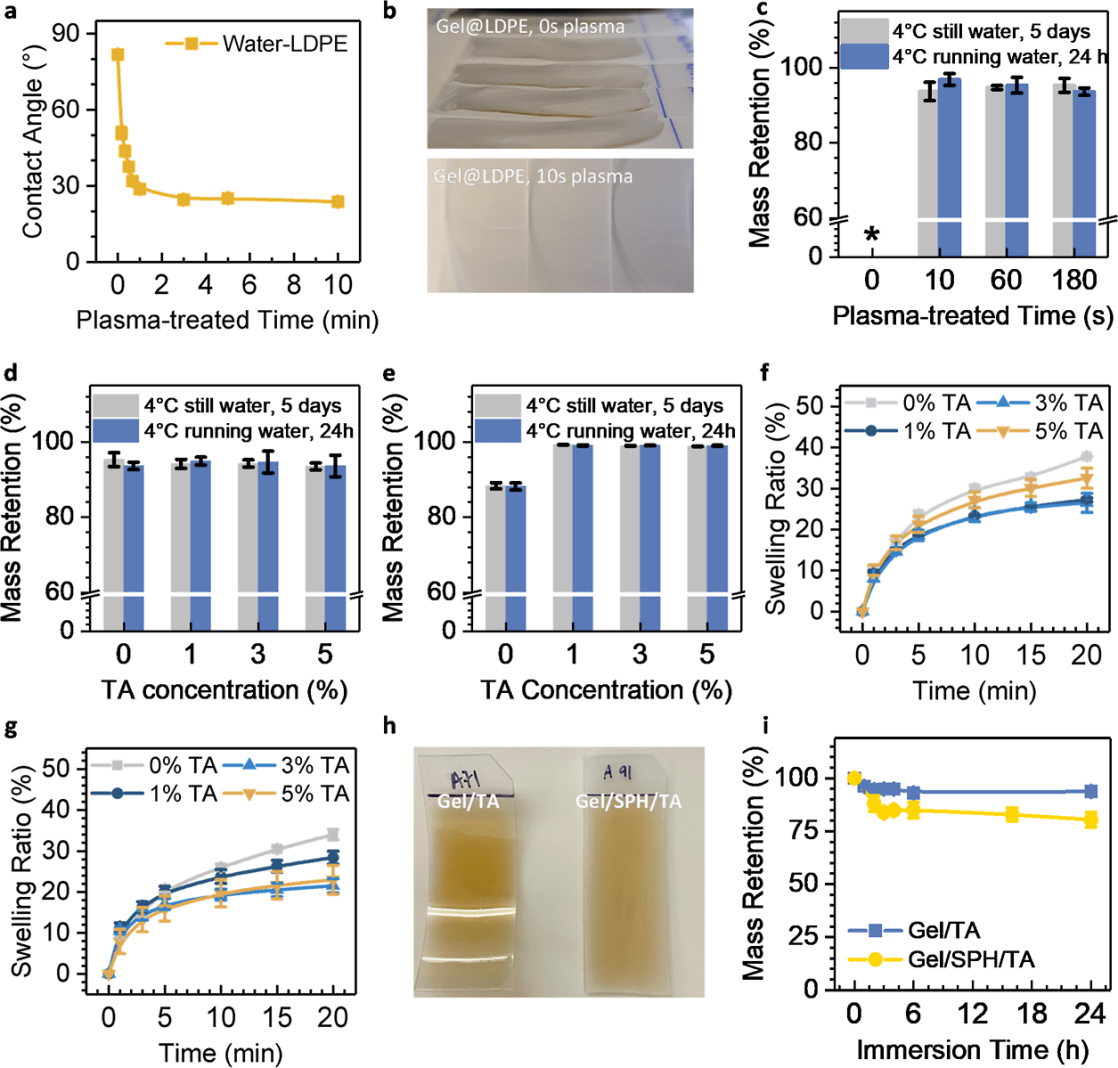
(a) Change of water contact angles of LDPE along with the increase in plasma treatment duration. (b) Images of Gel@LDPE coating-loaded LDPE coupons with (bottom) or without (top) 10 s plasma treatment. (c) Mass changes of the Gel@LDPE coupons after immersion in a still water bath or shaking water bath at 4 °C. (d,e) Mass retention ratios of Gel-based (d) and Gel/SPH-based (e) coating systems with different TA concentrations on LDPE coupons after immersion in a still water bath or shaking water bath at 4 °C. (f,g) Swelling ratios of Gel-based (f) and Gel/SPH-based (g) coating systems with various TA concentrations under ambient conditions. (h) Appearances of Gel/TA@LDPE and Gel/SPH/TA@LDPE-deposited LDPE coupons. (i) Mass retention rate of Gel/TA@LDPE and Gel/SPH/TA@LDPE systems after immersion in an ambient-condition water bath. The plotted data represent the means ± SD of three replicates.

The atmospheric plasma treatment was used to modify the inherent hydrophobic nature of LDPE and facilitate the subsequent deposition and interaction of protein coatings with LDPE. Contact angle measurements were taken and are shown in Figures 2a and S1, revealing a sharp reduction of water contact angles on the treated LDPE within the first minute of plasma treatment, reaching equilibrium after 3 min. The thickness of the protein-based coating is vital in indicating the overall charging capacity and potential antimicrobial performance. For consistent coating with uniform thickness, a pipetting method was employed to precisely control the pick-up rate, with 500 μL of the protein solution (10% total protein content) at 50 °C evenly spread over a 20 × 50 mm LDPE coupon (subject to plasma-treatment for 3 min). Due to their reduced viscosity at higher temperatures, the warm protein solutions naturally spread out evenly. As demonstrated in Figure S2, the mass deposition rates of the coatings were maintained at 7.5 ± 0.5% relative to the mass of LDPE coupons, ensuring uniform coatings on the coupons prepared for further analysis. Simulating the application conditions, the stability of various coating systems was evaluated with fully dried, coated LDPE coupons subjected to different aqueous immersion environments.

The enhanced surface hydrophilicity of the plasma-treated LDPE led to improved adhesion of protein coatings to LDPE surfaces, as demonstrated in Figure 2b. The protein coating exhibited inadequate adhesion on the untreated LDPE coupons but stable adhesion on the LDPE after 10 s of plasma treatment. This finding is supported by the results presented in Figure 2c, which indicate that 10 to 180 s of the plasma treatment provided a consistent and stable retention rate of protein deposition rates on LDPE coupons, capable of withstanding immersion in a 4 °C still water bath for 5 days or rinsing in a 4 °C still water bath for 24 h. Conversely, without plasma treatment, the protein-coated layer was rapidly peeled off upon water immersion. To ensure optimal stability of the deposition layer, a 3 min plasma treatment duration was employed in all subsequent deposition treatments.

To enhance the stability of the water-soluble protein-based coating system in aqueous environments, TA, a food-grade natural agent, was utilized as a crosslinking agent for the proteins. TA crosslinks proteins through physical and chemical mechanisms.^25^ Physical crosslinking involves hydrogen bonding and π–π stacking with the benzene rings in phenylalanine (Phe), tyrosine (Tyr), and tryptophan (Trp). Covalent bond formation between TA and proteins is pH-dependent and oxygen-sensitive, with TA transitioning from a phenol to a quinone structure at pH 8, enabling Michael addition and Schiff base reactions with amine groups in protein chains, as depicted in Figure 1b. The impact of TA on the stability of the protein coating was more pronounced in the Gel/SPH system, as illustrated in Figures 2d,e, where the mass retention rates of Gel-based coating systems remained unchanged regardless of the presence of TA, while the incorporation of TA in a Gel/SPH-based coating system was crucial in reducing mass loss in aqueous environments. Due to its higher degree of hydrolysis, SPH has a higher solubility in water with a small molecular size.^26^Figure 2e illustrates that the incorporation of 1% TA, based on total protein content, led to a significant enhancement in the stability of the Gel/SPH-based coating system on plasma-treated LDPE coupons. However, increasing the TA concentration beyond this level did not yield a significant improvement in the stability of protein systems.

The swelling ratios of Gel-based and Gel/SPH-based coating systems with different TA concentrations revealed the degree of crosslinking, as shown in Figures 2f,g. The addition of 1% TA in both systems increased their degrees of crosslinking and reduced swelling ratios, consistent with stability test results. However, increasing the concentration of TA beyond 1% did not further increase the crosslinking degree. Instead, when 5% TA was added, the swelling speed and swelling ratio of Gel/TA coating slightly increased, indicating less effective crosslinking compared to 1–3% TA, possibly due to potential TA aggregation. Gel/SPH-based systems with 3 and 5% TA exhibited similar swelling behaviors, indicating that 3% TA was efficient enough to crosslink, as described in Gel/SPH-based deposition systems.

The swelling test results indicate that the addition of 1% TA improved the crosslinking degree of both Gel-based and Gel/SPH-based coating systems. The increase in crosslinking degree is more important to Gel/SPH-based systems, as they exhibit less stability compared to Gel-based systems. To ensure a fair comparison of the two systems’ performance, 1% TA was added to both Gel-based and Gel/SPH-based coating systems, resulting in Gel/TA@LDPE (10% gelatin with 1% TA, TA concentration calculated based on total protein content) and Gel/SPH/TA@LDPE (9% gelatin, 1% SPH with 1% TA, TA concentration calculated based on total protein content) for subsequent analysis. A photo image with both Gel/TA@LDPE and Gel/SPH/TA@LDPE is presented in Figure 2h, revealing the appearance of the coated LDPE coupons. The stability of Gel/TA@LDPE and Gel/SPH/TA@LDPE was further tested in an ambient-temperature water bath, and the mass retention rates were tested and are shown in Figure 2i. The results showed that Gel/TA@LDPE retained 94.6% and 93.8% of the initial deposition mass, while Gel/SPH/TA@LDPE retained 88.2% and 80.4% of the initial deposition mass after 2 and 24 h of water immersion, respectively. The overall mass retention was satisfactory, considering that the coating systems are not expected to function in ambient-temperature aqueous solutions for extended periods in the application scenario of totes, sorting tables, packaging equipment, storage racks, or other hydrophobic plastic surfaces in the fresh-produce processing facility. Both deposition systems remained stable with short-term exposure to ambient water.

In summary, plasma treatment of LDPE surfaces enhanced interactions between the plastic and protein coating systems, and the use of TA increased the stability of the protein-based coating systems in aqueous solutions. The Gel/TA@LDPE and Gel/SPH/TA@LDPE coating systems demonstrated stable performance in long-term chilled water immersion and short-term ambient water immersion and have the potential to function as rechargeable halamine biocidal systems.

### 2.2 Rechargeable Chlorination of the Gel/TA@LDPE and Gel/SPH/TA@LDPE

The developed Gel/TA@LDPE and Gel/SPH/TA@LDPE coatings can be efficiently chlorinated to form biocidal halamine structures of Gel/TA-Cl^+^@LDPE and Gel/SPH/TA-Cl^+^@LDPE by immersing the coated LDPE coupons in a diluted chlorination solution. However, it is noteworthy that the intrinsic susceptibility of proteins to oxidative free chlorine limits the use of highly concentrated chlorination solutions, despite their ability to rapidly charge the available halamine precursors. To better control the chlorination process and minimize protein oxidation, chlorination solutions containing 10 or 20 ppm of free active chlorine content were utilized to charge the Gel/TA@LDPE-coated or Gel/SPH/TA@LDPE-coated LDPE coupons. The active chlorine contents of chlorinated deposition systems were measured using an established iodometric titration method.^17^

**Figure 3:**
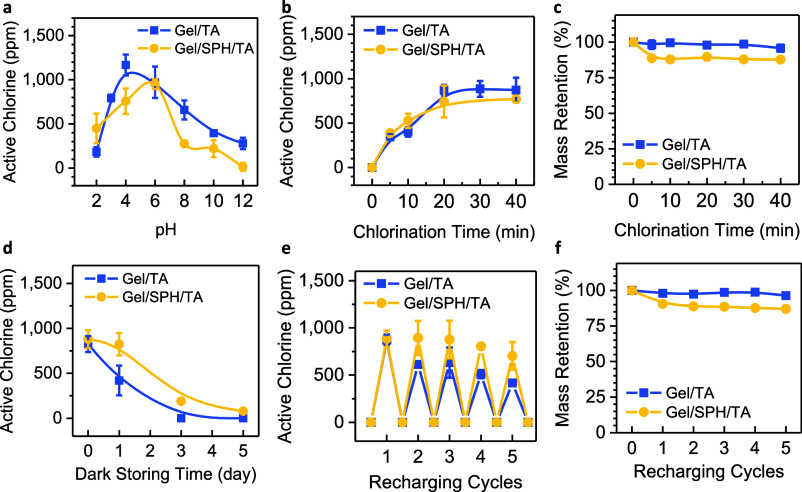
(a,b) Active chlorine contents of Gel/TA@LDPE (Gel/TA-Cl^+^@LDPE) and Gel/SPH/TA@LDPE (Gel/SPH/TA-Cl^+^@LDPE) after being charged for 1 h in chlorination solution with 20 ppm free active chlorine at different pH levels (a), and after being charged in chlorination solution with 20 ppm free active chlorine at pH 6 for various duration (b). (c) Mass retention rates of Gel/TA@LDPE (Gel/TA-Cl^+^@LDPE) and Gel/SPH/TA@LDPE (Gel/SPH/TA-Cl^+^@LDPE)when charged in chlorination solution (pH6, 20 ppm free active chlorine) for various duration. (d) Stability of Cl^+^-charged deposition systems against storage time at 21 °C in the dark. (e,f) Active chlorine content (e) and mass retention rates (f) of Gel/TA@LDPE and Gel/SPH/TA@LDPE coating systems over multiple chlorination cycles, with each cycle comprising a charging step of a 20 min incubation in the chlorination solution (pH6, 20 ppm free active chlorine) and a quenching step of a 10 min incubation in 0.001 N sodium thiosulfate solution. In all the legends, Gel/TA@LDPE and Gel/TA-Cl^+^@LDPE were marked as Gel/TA, and Gel/SPH/TA@LDPE and Gel/SPH/TA-Cl^+^@LDPE as Gel/SPH/TA for brevity. The plotted data are expressed as the means ± SD of three replicates.

The chlorination efficiency was significantly influenced by the pH conditions of the solutions due to the varying reactivity of hypochlorous moieties (HOCl/OCl^–^) with amine/amide structures.^12^ In the tests involving different pH conditions, 100 mL chlorination solutions with 10 ppm of free active chlorine content (total available active chlorine content at 2000 ppm for each coated specimen) were employed across a wide range of pH values. At low pH values, the amino precursor groups in the proteins (p*K*_a_ at 7.5 to 8.0), the side chain amino groups of lysine residues (p*K*_a_ of 10.5) and the hypochlorous acid (p*K*_a_ of 7.53) can be protonated, forming structures of –NH_3_^+^ and HOCl. The protonated primary amines are less likely to be converted to *N*-halamine structures (NH–Cl), while HOCl is more effective than ClO^–^ in generating *N*-halamine structures.^15^ However, the abundant peptide (amide) structures in proteins could react with hypochlorous acid. Consequently, Gel/TA@LDPE exhibited the highest ability in forming *N*-halamine structures, Gel/TA-Cl^+^@LDPE, at pH 4, with a subsequent decrease in total active chlorine content from pH 4 to pH 12, as shown in Figure 3a. Similarly, Gel/SPH/TA@LDPE achieved the most efficient *N*-halamine formation into Gel/SPH/TA-Cl^+^@LDPE at pH 6, followed by a reduction in total active chlorine content from pH 6 to pH 12. Both Gel/SPH/TA@LDPE and Gel/TA@LDPE showed optimal pH in acidic conditions with minor differences. The minor differences could be contributed by the presence of various functional groups between gelatin and SPH and their different interactions with chlorine at different pH levels.

When designing the coating systems, both the chlorination efficiency and safety considerations related to food handling and operational aspects of the application environment should be considered.^27^ Thus, a relatively mild yet efficient chlorination condition at pH 6 was chosen for the chlorination solutions of both Gel/TA@LDPE and Gel/SPH/TA@LDPE deposition systems to achieve a satisfying chlorination level.

The total amount of active chlorine in the charged *N*-halamine structure also depends on the chlorination time. In experiments monitoring *N*-halamine formation from Gel/TA@LDPE and Gel/SPH/TA@LDPE to Gel/TA-Cl^+^@LDPE and Gel/SPH/TA-Cl^+^@LDPE over time, chlorination solutions with 20 ppm free active chlorine at pH 6 were employed to facilitate charging efficiency in a food-safety-appropriate environment. Figure 3b demonstrates the active chlorine content of Gel/TA-Cl^+^@LDPE and Gel/SPH/TA-Cl^+^@LDPE deposition systems after immersion in the chlorination solutions (20 ppm active chlorine content, pH 6) for periods up to 40 min. Both systems exhibited an increase in the active chlorine content over chlorination time. The formation of both Gel/TA-Cl^+^@LDPE and Gel/SPH/TA-Cl^+^@LDPE reached equilibrium in approximately 20 min, with active chlorine contents of 871 ± 60 and 742 ± 179 ppm, respectively. These two systems demonstrated similar charging performance and comparable maximum capacity in the chlorination solution (20 ppm active chlorine content, pH 6). Compared to previously reported materials with effective antimicrobial properties, both coating systems exhibited high active chlorine capacities, indicating the potential antimicrobial capabilities of the developed active chlorine-charged coating systems, Gel/TA-Cl^+^@LDPE and Gel/SPH/TA-Cl^+^@LDPE.^12,15−17^

The maximum charging capacity of the Gel/TA@LDPE and Gel/SPH/TA@LDPE coating systems for *N*-halamine formation depends on the amount of accessible amino and peptide bonds in proteins. An increase in accessibility to the precursor groups leads to a more active chlorine reservoir. SPH, in contrast to gelatin, has a significantly lower molecular weight as a highly hydrolyzed protein with a higher solubility in water. The increased water solubility of uncrosslinked SPH in the systems contributes to the slight weight losses of Gel/SPH/TA@LDPE, as shown in Figures 2i and 3c. The loss of SPH in the systems could increase the access of the remaining deposition to chlorinating agents. Considering the potential instability of protein-based deposition systems in oxidative environments, we also measured the mass retention of both coating systems in the aforementioned chlorination solution (20 ppm active chlorine content, pH 6). Figure 3c indicates that Gel/TA@LDPE (or Gel/TA-Cl^+^@LDPE) and Gel/SPH/TA@LDPE (or Gel/SPH/TA-Cl^+^@LDPE) lost only small percentages of mass, less than 5% and 14%, respectively, after immersion in the described chlorination baths for 40 min. The mass losses of the two coating systems during chlorination were slightly higher compared to the mass loss in chlorine-free water under ambient conditions, as depicted in Figure 2i. This confirmed that the employed chlorination solution with a 20 ppm active chlorine content at pH 6 could still provide a suitable charging environment without significantly impacting the capacity of the two coating systems. Furthermore, the Gel/TA-Cl^+^@LDPE and Gel/SPH/TA-Cl^+^@LDPE showed potential as rechargeable antimicrobial systems, as the coating masses were maintained during the charging process.

The stability of the two charged systems, Gel/TA-Cl^+^@LDPE and Gel/SPH/TA-Cl^+^@LDPE, was assessed by storing the coated LDPE coupons in a dark environment at 21 °C with 40% relative humidity for up to 5 days. To ensure a fair comparison, both coating systems were charged with chlorination solutions (20 ppm, pH 6) for 20 min, reaching an active chlorine content of 850 ± 30 ppm at day 0. Figure 3d reveals that Gel/SPH/TA-Cl^+^@LDPE retained more active chlorine over the storage time compared to the Gel/TA-Cl^+^@LDPE systems. The active chlorine content of Gel/SPH/TA-Cl^+^@LDPE decreased from 879 (day 0) to 824 ppm after 1 day, 190 ppm after 3 days, and 78.4 ppm after 5 days. In contrast, the active chlorine content of Gel/TA-Cl^+^@LDPE decreased from 826 ppm (day 0) to 420 ppm after 1 day and fell below the detection limit after 3 days. The stability of both Gel/TA-Cl^+^@LDPE and Gel/SPH/TA-Cl^+^@LDPE appeared limited at ambient conditions beyond 1 day of storage. Consequently, it is recommended to charge both deposition systems immediately prior to their intended use for optimal efficiency. Additionally, reducing the storage temperatures may enhance their storage stability, given the temperature-dependent nature of the halamine structures.

Gel/SPH/TA-Cl^+^@LDPE exhibited better storage stability than Gel/TA-Cl^+^@LDPE in the tests, potentially due to differences in their composition or structure, which might lead to different interactions with active chlorine and/or more stable retention of active chlorine within the Gel/SPH/TA-Cl^+^@LDPE system. During chlorination, both the Gel/TA@LDPE and Gel/SPH/TA@LDPE systems swell, facilitating the penetration of HOCl molecules into the protein-based coating layers to charge any accessible sites. However, in the Gel/SPH/TA-Cl^+^@LDPE system, the dissolution of uncrosslinked SPH molecules could increase the accessibility of charging sites throughout the thickness of the coating layer, exposing more gelatin for chlorination. Conversely, for Gel/TA@LDPE, the chlorination predominantly occurs on the surface due to its more solid coating structure. As the charged systems are air-dried, surface-charged active chlorine in Gel/TA-Cl^+^@LDPE can be rapidly released through interactions with air moisture, whereas the structure of Gel/SPH/TA-Cl^+^@LDPE acts as a reservoir, forming “storage cells” beneath the coating top surface and maintaining a higher concentration of active chlorine over time.

Moreover, the rechargeability of both Gel/TA@LDPE and Gel/SPH/TA@LDPE coating systems was demonstrated through repeated chlorination and quenching cycles, as illustrated in Figure 3e. Each cycle involved a 20 min chlorination step followed by a 10 min quenching step in a thiosulfate solution. This cycle was repeated five times. The active chlorine contents of Gel/TA-Cl^+^@LDPE decreased from 871 ppm in the first cycle to 417 ppm by the fifth cycle, whereas Gel/SPH/TA-Cl^+^@LDPE retained a more stable active chlorine level, from 885 ppm in the first cycle to 704 ppm in the fifth cycle.

The sustained rechargeability of both coating systems depends on both their stable chemical and coating structures under ambient and low-temperature conditions. Figure 3f presents the mass retention of the deposition systems throughout five recharging cycles. The Gel/TA@LDPE coating system maintained over 96% of its initial deposition mass after five charging–quenching cycles, indicating its considerable stability. Gel/SPH/TA@LDPE showed less stability as illustrated in previous tests and retained more than 87% of its initial deposition mass after 5 charging–quenching cycles. It is noteworthy to state that repeated charging–quenching cycles did not induce a consistent decline in the deposition mass, as the mass reduction was primarily induced by the first charging cycle for both Gel/TA@LDPE and Gel/SPH/TA@LDPE coatings, agreeing with what was found in Figure 3c.

An interesting observation was that Gel/TA@LDPE showed less mass reduction but a greater active chlorine loss. The observed rechargeability of Gel/TA@LDPE and Gel/SPH/TA@LDPE coatings partially relies on the mass retention of the coating materials. As previously discussed, the high solubility and subsequent dissolution of uncrosslinked SPH during the repeated charging process in the Gel/SPH/TA@LDPE system may expose more internal sites for chlorination. This structural feature amplifies the contact area between the coating and the charging solution, thereby accelerating the recharging rate in the Gel/SPH/TA@LDPE system in comparison with the Gel/TA@LDPE system.

The findings indicate that a chlorination condition of 20 ppm of free active chlorine at a pH of 6 is well-suited for these protein-based coating systems. Consequently, the resulting charged Gel/TA-Cl^+^@LDPE and Gel/SPH/TA-Cl^+^@LDPE coatings could effectively function on food-contact surfaces, offering promising rechargeable biocidal properties.

### 2.3 Antimicrobial Performances of Gel/TA-Cl^+^@LDPE and Gel/SPH/TA-Cl^+^@LDPE

The antimicrobial capabilities of the Gel/TA-Cl^+^@LDPE and Gel/SPH/TA-Cl^+^@LDPE coating systems were subsequently assessed. For a balanced comparison of their antimicrobial effects, both systems were activated in a diluted chlorination solution containing 20 ppm of active chlorine at pH 6 for 20 min, generating the active forms Gel/TA-Cl+@LDPE and Gel/SPH/TA-Cl+@LDPE coated on the LDPE coupons. Both systems exhibited active chlorine content in the vicinity of 850 ± 30 ppm. Their antimicrobial effectiveness was evaluated against both *Escherichia coli* (*E. coli*, Gram-negative) and *Listeria innocua* (*L. innocua*, Gram-positive) to test their contact killing performances.

**Figure 4:**
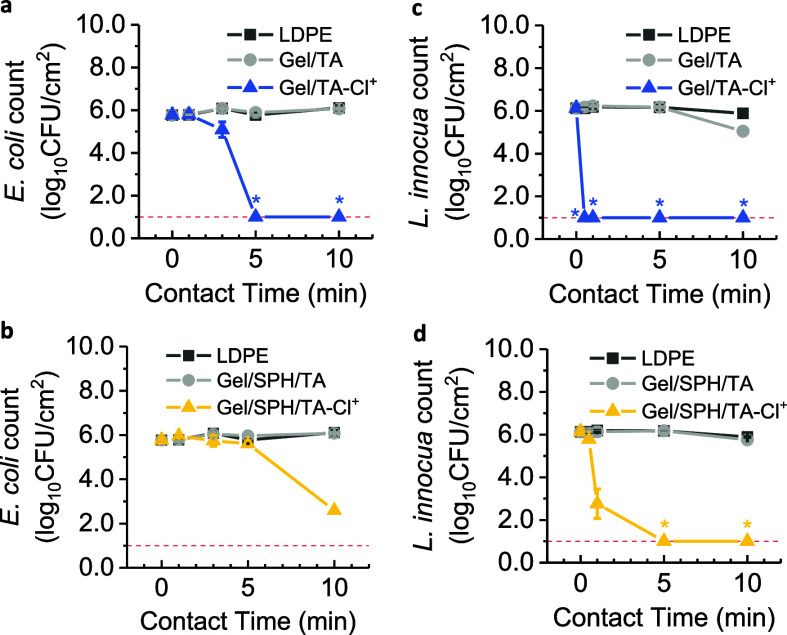
Contact-killing efficiency of Gel/TA-Cl^+^@LDPE (a and c) and Gel/SPH/TA-Cl^+^@LDPE (b and d) against *E. coli* (a and b) and *L. innocua* (d and d), respectively. Each charged coupon had an active chlorine content of 850 ± 30 ppm. The detection limit for the bacterial count was 1 log_10_cfu·cm^–2^, which was indicated by red dotted lines in the graphs. Star symbols denote instances where the bacterial count fell below the detection limit. The data presented are the mean ± SD of three replicates.

As depicted in Figure 4a,b, the initial concentration of *E. coli* on inoculated coupons was approximately 5.8 ± 0.1 log_10_cfu·cm^–2^. A reduction of viable *E. coli* counts (5.1 ± 0.4 log_10_cfu·cm^–2^) was observed after 3 min of contact with Gel/TA-Cl^+^@LDPE coupons, outperforming both the uncoated control (LDPE, 5.8 ± 0.1 log_10_cfu·cm^–2^) and the uncharged coated control (Gel/TA @LDPE, 5.8 ± 0.1 log_10_cfu·cm^–2^). The *E. coli* concentration dropped below 1.0 log_10_cfu·cm^–2^ after 5 min of contact with Gel/TA-Cl^+^@LDPE, translating to a 99.998% reduction within this time frame. Similar efficacy was noted for the Gel/SPH/TA-Cl^+^@LDPE systems, where the viable *E. coli* dropped from 5.8 ± 0.1 to 2.6 ± 0.1 log_10_cfu·cm^–2^ within a 10 min contact period. This resulted in a 99.94% decrease within 10 min relative to both the uncoated (LDPE) and coated but uncharged controls (Gel/SPH/TA@LDPE). The relatively slower killing speed of Gel/SPH/TA-Cl^+^@LDPE systems is possibly caused by less active chlorine on the outside surface of the coated system, consistent with the structural features.

As illustrated in Figure 4c,d, both Gel/TA-Cl^+^@LDPE and Gel/SPH/TA-Cl^+^@LDPE systems exhibited accelerated contact-killing effects against *L. innocua* compared to *E. coli*. In particular, the Gel/TA-Cl^+^@LDPE system lowered the L. innocua concentration from 6.1 ± 0.1 log_10_cfu·cm^–2^ to below 1.0 log_10_cfu·cm^–2^, achieving a 99.999% reduction within a 1 min contact period. Gel/SPH/TA-Cl^+^@LDPE demonstrated comparable efficacy, reducing the *L. innocua* concentration from 6.1 ± 0.1 to 2.8 ± 0.7 log_10_cfu·cm^–2^ within 1 min and to less than 1.0 log_10_cfu·cm^–2^ after 5 min, also resulting in a 99.999% reduction.

Although further detailed research should be deployed, the contact-killing tests showcased in the study reveal that the developed coating systems, both Gel/SPH/TA-Cl+@LDPE and Gel/TA-Cl+@LDPE systems, possess a substantial capability to reduce or prevent biofilm formation by effectively reducing the bacterial count on surfaces.^18^

### 2.4 Temperature-sensitive Detachable Coating Design and Antifouling Performance

**Figure 5:**
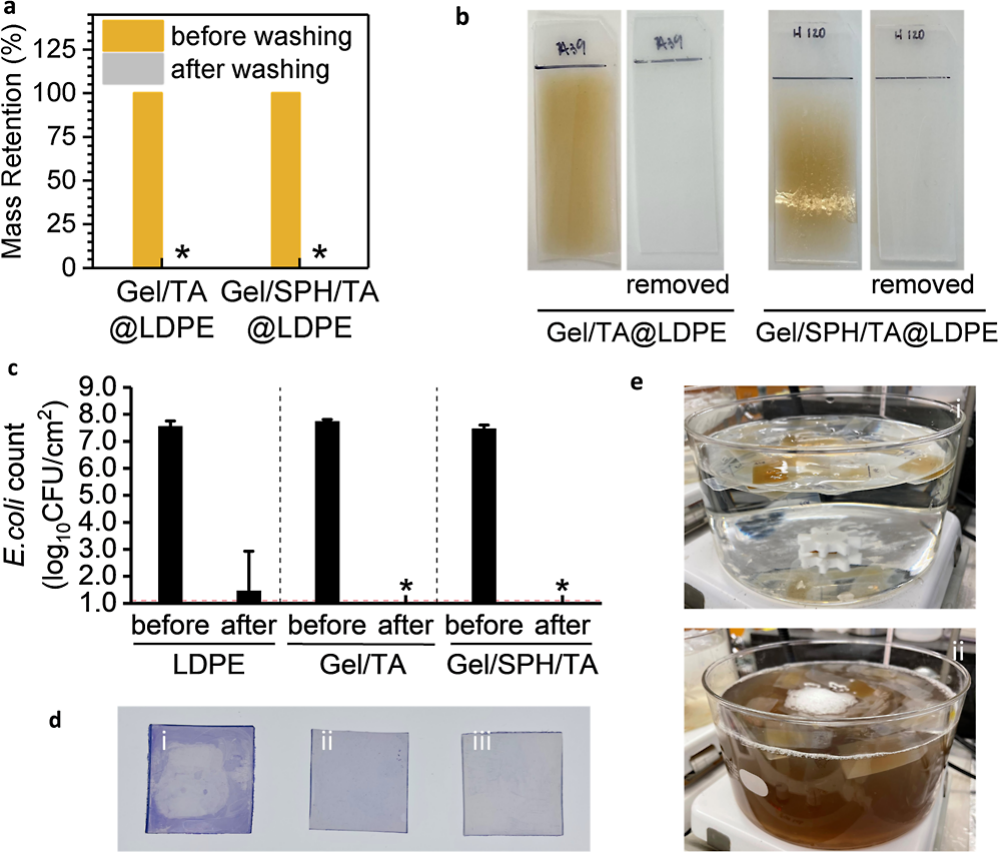
(a) Effectiveness of 50 °C water in removing the deposition systems from LDPE coupons, with star symbols indicating instances where the postwash mass fell below the detection limit. (b) Images of LDPE coupons before and after undergoing the hot water rinse. (c) *E. coli* planktonic cell counts on LDPE coupons after 4 days of biofilm cultivation, both prior to and subsequent to hot water treatment; a detection limit of 1.1 log_10_cfu·cm^–2^ was marked with a red dotted line, with star symbols highlighting results beneath this limit. (d) Untreated (i), Gel/TA@LDPE-coated, and Gel/SPH/TA@LDPE-coated (iii) LDPE coupons, all of which were submerged in an *E. coli* suspension for 4 days, subjected to a 50 °C hot water treatment for 3 min, and then stained with a 0.1% crystal violet aqueous solution. (e) Condition of the 50 °C hot water bath before (i) and after (ii) the cleansing of 250 pieces of Gel/TA@LDPE- and Gel/SPH/TA@LDPE-coated LDPE coupons. The plotted data are expressed as the means ± SD of three replicates.

The developed sustainable biocidal coating systems, as demonstrated in Figure 1a, are designed to inhibit biofilm formation on hydrophobic food-contact surfaces through the implementation of a rechargeable *N*-halamine structure alongside a detachable coating design. After each use cycle, the protein-based antimicrobial layers—Gel/SPH/TA@LDPE or Gel/TA@LDPE— can be easily removed during routine sanitation procedures. By carefully controlling the degree of crosslinking, both Gel/TA@LDPE and Gel/SPH/TA@LDPE demonstrated stable performance under chilled or ambient conditions while ensuring ease of removal with hot water. In this study, a 50 °C water bath was used to remove the protein coating, a temperature that needs low energy to achieve and is easy to manage by fresh produce processors. As illustrated in Figure 5a,b, immersing the coated LDPE coupons in a 50 °C water bath for two min with slight agitation allowed the coatings to be completely washed away. This process effectively returns the LDPE coupons to their original state, highlighting the coatings’ suitability for applications where regular sanitation is crucial and the ease of cleaning is a significant advantage.

The contact killing performance already demonstrated the potential for preventing biofilm formation. However, if minor biofilms form during fresh produce processing due to various factors, then the coating detachment step can efficiently eliminate these formed biofilms. As demonstrated in Figure 5c,d, biofilms of *E. coli* were intentionally cultivated on both uncoated and coated (Gel/TA@LDPE double-sided coated, or Gel/SPH/TA@LDPE double-sided coated) LDPE coupons. The initial viable counts of uncoated, Gel/TA@LDPE double-sided coated, and Gel/SPH/TA@LDPE double-sided coated LDPE coupons were 7.7, 7.7, and 7.5 log_10_cfu·cm^–2^, respectively. Upon immersing these biofilm-laden coupons in a 50 °C water bath and subsequently rinsing, it was observed that the detachment of protein-based coatings facilitated the complete removal of the developed biofilms, as depicted in Figure 5c.

The effectiveness of the coating removal and biofilm eradication was further validated through crystal violet (CV) assays, which provided visual evidence of the significant reduction in biofilm residues post-treatment, as demonstrated in Figure 5d. CV, a cationic dye, binds to the polysaccharides, proteins, and nucleic acids in cells and the matrix of extracellular polymeric substances in the biofilms, as well as the protein residues from the deposition systems, providing a visible stain.^28,29^ This contrast was particularly noticeable when comparing the coated coupons (both Gel/TA@LDPE and Gel/SPH/TA@LDPE), which showed minimum residual biofilms or coating layers, to the uncoated coupons, which displayed significant biofilm remnants.

In addition, the two designed coating systems ensured efficient detachment with minimal water consumption. As evidenced in Figure 5e, immersing the coated LDPE coupons in a stirred bath of 50 °C water for 1 min successfully removed both the Gel/TA@LDPE and Gel/SPH/TA@LDPE coatings. The findings also imply that the application of pressurized steam could potentially remove the depositions even more swiftly and effectively. Importantly, the removal of these coatings from LDPE necessitates only a limited volume of water, with 1 L of 50 °C water shown to process over 250 coated LDPE coupons (Figure 5e). The resultant wastewater can be disposed of directly through the sewage system, given that all components of the coatings are environmentally friendly and biodegradable.

In conclusion, the Gel/TA@LDPE and Gel/SPH/TA@LDPE deposition systems have demonstrated robust performances at and below ambient conditions while also being easily removable by hot water or steam. These sustainable and environmentally friendly coating systems have the potential to provide effective and safe antimicrobial protection for different hydrophobic food-contacting surfaces. This study demonstrates a new technique that can effectively reduce the formation of biofilms on surfaces of materials with less impact on the environment. The overall process is doable without much technical barriers, as the atmosphere plasma treatment can be flexibly employed on most surfaces and paint coating is easy to practice. The results can trigger more interest from researchers and processors to develop and adopt more environmentally friendly techniques.

## 3 Conclusions

In this study, we have successfully developed Gel/TA@LDPE and Gel/SPH/TA@LDPE as rechargeable, removable antimicrobial, and antifouling coatings for hydrophobic food-contact surfaces. These systems effectively combat foodborne bacterial contamination via *N*-halamine biocidal structures. The robust antimicrobial and antifouling performance of the coatings prevent biofilm formation and efficiently eliminates existing bacterial colonies. Specifically, the Gel/SPH/TA@LDPE coating system featured higher capacity and stability in forming *N*-halamine structures by offering a higher surface area for chlorine charging, allowing efficient diffusion of antimicrobial agents, and enhancing storage stability and antimicrobial capabilities. The protein coating systems enable multiple chlorine recharging cycles under mild conditions (chlorination solution with 20 ppm of active chlorine at pH 6) and easy removal with hot water or steam, aligning with current sanitation procedures in the fresh produce industry. The design of the removable coating systems not only addresses the operational efficiency and microbial safety concerns associated with fresh produce processing but also aligns with broader environmental sustainability goals. Overall, Gel/TA@LDPE and Gel/SPH/TA@LDPE systems offer a sustainable, effective, and practical solution for improving food safety, making them promising candidates for widespread implementation in food processing settings.

## 4 Experimental Section

### 4.1 Materials

Gelatin powder (type A, 300 bloom), SPH, sodium hydroxide, hydrochloric acid, methanol, ethyl acetate, hexane, acetone, sodium chloride, potassium chloride, sodium phosphate dibasic, potassium phosphate monobasic, hydrochloric acid, glucose, M9 minimal salts (5×), and CV solution (1%) were purchased from Sigma-Aldrich (Milwaukee, WI). TA was purchased from Alfa Aesar (Thermo Fisher, Belgium). Anhydrous sodium thiosulfate, 0.1 N iodine standard solution, and 0.1 N sodium thiosulfate standard solution were purchased from VWR Chemicals (Radnor, PA). Tryptic soy broth (TSB) was purchased from Neogen (Lansing, MI), and tryptic soy agar (TSA) was purchased from bioWorld (Dublin, OH). Tween 20 was purchased from ChemImpex (Wood Dale, IL). Rifampicin was purchased from Thomas Scientific LLC (NJ, USA). Tryptone was purchased from Amresco (Solon, OH). Clorox bleach solution with a free chlorine content of 8.0% was produced by Clorox Co., Ltd. (Oakland, CA, USA). LDPE sheets were purchased from the Henta Corporation. DI water was used in the materials fabrication and tests.

### 4.2 Preparation of Antimicrobial Coating

Three homogeneous stock solutions, gelatin–water (15%), SPH – water (10%), and TA – water (10%), were prepared in advance. Biomass-based coating solutions were made by appropriately mixing gelatin stock solution (15%), SPH stock solution (10%), TA stock solution (10%), and DI water at 50 °C and adjusting the pH to 8 using a diluted sodium hydroxide solution. In the Gel/TA coating formulations, 10% gelatin was crosslinked with 1, 3, or 5% TA. For the Gel/SPH/TA coatings, a combination of 9% gelatin and 1% SPH was crosslinked using 1, 3, or 5% TA. All prepared solutions were directly used or stored at 4 °C for preservation. LDPE sheets were cut into 20 × 50 mm rectangle coupons. Before coating, the LDPE coupons were subjected to a cleaning procedure and plasma treatment, consecutively. The cleaning procedure includes consecutive baths of DI water, methanol, ethyl acetate, hexane, and acetone to eliminate any surface dirt, moisture, and oil components. The plasma treatment was delivered using a plasma cleaner (Harrick Plasma, NY 14850) accompanied by a Super Evac vacuum pump (model 93560) for various amounts of time. Each cleaned LDPE coupon (20 × 50 mm) was evenly coated with 500 μL of Gel/TA solution or Gel/SPH/TA coating solutions using a silicone scraper to make Gel/TA@PE or Gel/SPH/TA@PE. The coated coupons were fully dried under ambient conditions, transferred, and stored in a desiccator with calcium chloride to dehydrate before mass measurement.

### 4.3 Water Contact Angle

The LDPE coupons were treated by plasma using the Plasma Cleaner (Harrick Plasma, NY 14850) accompanied by a Super Evac vacuum pump (model 93560) for various amounts of time. The contacting angle between water and LDPE coupons (plasma-treated or untreated) was observed and analyzed by a Dino-Lite digital microscope (Dunwell Tech. Inc., Torrance, CA).

### 4.4 Mass Retention Rate

The mass retention of various coating systems on the LDPE was tested in a 4 °C still water bath, a 4 °C shaking water bath, a 21 °C still water bath, and a 21 °C chlorination bath (10–100 ppm), respectively. In each test, one deposited PE coupon was immersed in 40 mL of bath liquid and incubated for the desired time under certain conditions. The post-treated coupons were fully dried in a desiccator at ambient conditions until constant mass was obtained. The mass retention rate was calculated according to eq 1, where *m* is the dry weight of the post-treated deposited coupon in g, *m*_0_ is the initial dry weight of the LDPE coupon before deposition in g, and *m*_1_ is the dry weight of the untreated deposited coupon in g.
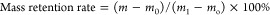
1

### 4.5 Swelling Ratio

The evaluation of the swelling ratio was conducted following a protocol described by Zou et al., utilizing eq 2. This equation calculates the ratio based on the specimen’s weight postimmersion in a water bath for a predetermined period, denoted as *m*, against the specimen’s initial weight, *m*_o_.^30^
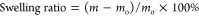
2

### 4.6 Chlorination and Active Chlorine Content

A chlorination solution containing 10 or 20 ppm of active chlorine was prepared by diluting a commercial sodium hypochlorite solution. The pH conditions of the chlorination solutions were adjusted by diluted hydrochloric acid or sodium hydroxide solutions. In a typical chlorination step, one Gel/TA@LDPE coupon or Gel/SPH/TA@LDPE coupon was fully immersed in 100 mL of chlorination solution for the desired time with agitation. The chlorinated coupon was then rinsed with an excessive amount of DI water to remove free active chlorine. An established iodometric titration method was adopted and modified to quantify the active chlorine content of the Gel/TA-Cl^+^@LDPE or Gel/SPH/TA-Cl^+^@LDPE.^17^ Typically, one charged coupon was first fully quenched in 15 mL of 0.001 N sodium thiosulfate standard solution (excessive), and the sodium thiosulfate residue in the solution was titrated against the 0.001 N iodine standard solution. The active chlorine content was calculated according to eq 3, where *V*_2_ (mL) is the consumed *I*_2_ volume with an uncharged sample (Gel/TA@PE or Gel/SPH/TA@PE), *V*_1_ (mL) is the consumed *I*_2_ volume with charged samples (Gel/TA-Cl^+^@PE or Gel/SPH/TA-Cl^+^@PE), *N* = 10^–6^ mol·mL^–1^, and *W* is the coating mass in g.
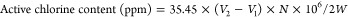
3

### 4.7 Bacterial Cultures

A rifampin (Rif)-resistant strain of *E. coli* O157:H7 (*E. coli*, ATCC 700728) was cultured in TSB and incubated overnight at 37 °C to achieve the stationary phase cultures, obtaining an *E. coli* bacterial culture of 5 × 10^8^ cfu/mL (assessed by plate count). A Rif-resistant *L. innocua* mutant (*L. innocua*, ATCC 33090), provided by Trevor Suslow (University of California, Davis), was cultivated similarly until a concentration of 8 × 10^8^ cfu·mL^–1^ was reached (assessed by plate count). Both bacterium cultures were centrifuged at 3000*g* for 8 min and triple washed in 1 × PBS buffer (pH 7.4) before use. The bacterial suspensions were prepared in sterilized 1 × PBS buffer (pH 7.4) for the following tests. For plate counting cultures, TSA plates supplemented with 50 μg·mL^–1^ Rif (TSAR) were used.

### 4.8 Antimicrobial Assays Against *L. innocua* and *E. coli*

The antimicrobial activities of Gel/TA-Cl^+^@LDPE- and Gel/SPH/TA-Cl^+^@LDPE-coated LDPE (2 × 5 cm, single-side-coated) were assessed against Rif-resistant *E. coli* O157:H7 (ATCC 700728) and Rif-resistant *L. innocua* (ATCC 33090) mutants. In the contact-killing tests, single-side coated Gel/TA@LDPE and Gel/SPH/TA @LDPE coupons were charged in a chlorination solution (active chlorine content at 1000 ppm) or immersed for the same duration in water to obtain Gel/TA@LDPE hydrogel and Gel/SPH/TA@LDPE hydrogel-coated LDPE coupons. In a typical test, 10 μL of overnight bacterial culture suspensions (undiluted) were inoculated onto the treated-sided surface of the LDPE coupons and incubated at ambient conditions for different time durations (0–10 min) at room temperature. After inoculation and incubation, the specimen coupon was transferred to a sterilized 50 mL test tube containing 10 mL of sterilized detachment solution (1 × PBS buffer supplemented with 1% Na_2_S_2_O_3_ and 0.2% Tween 20). The test tube was vortexed vigorously for 1 min to fully recover the remaining bacterium on the LDPE coupons. The enumeration of the bacteria population was then performed by serial spread-plate dilution on TSAR plates. The bacterial population was assessed after incubation of the agar plates for 48 h at 37 °C and expressed as log cfu·cm^–2^. Coated and charged LDPE coupons, Gel/TA-Cl^+^@LDPE and Gel/SPH/TA-Cl^+^@LDPE, with 1000 ppm active chlorine content, were tested for their antimicrobial behaviors. Noncoated LDPE and uncharged Gel/TA@LDPE- and Gel/SPH/TA@LDPE-coated LDPE coupons were used as controls. The antibacterial assays were performed in triplicate (*n* = 3).

### 4.9 Biofilm Assay

The uncoated Gel/TA@LDPE- and Gel/SPH/TA-@LDPE-coated LDPE coupons (2 × 2 cm, double-side-coated) were assessed with Rif-resistant E. coli O157:H7 (ATCC 700728) mutants for biofilm-forming possibility and removing efficiency. In a 6-well plate, place 2 × 2 cm specimens (double-side-treated) in 3 mL of bacteria suspension (7 log cfu·mL^–1^, suspended in M9 broth) to fully immerse the coupons. The six well plates were incubated in the dark under ambient conditions for 4 days to develop biofilms. After 4 days, the coupons were recovered from the wells and gently rinsed with 10 mL of PBS buffer twice in the six well plate. The LDPE coupons with developed biofilms were either directly enumerated or subjected to hot water bath treatments before enumeration.

### 4.10 Removal Efficiency Test

To mimic the cleaning procedure, one LDPE coupon (coated or uncoated) with developed biofilms was first immersed in a 50 mL 50 °C water bath for 2 min with minor swinging and then rinsed in another 30 mL 50 °C water bath for 1 min to remove the remaining residue of coating. For enumeration, LDPE coupons were transferred to 50 mL centrifuge tubes with 10 mL detachment solutions (1 × PBS buffer supplemented with 1% Na_2_S_2_O_3_ and 0.2% Tween 20). The centrifuge tubes were vortexed for 2 min twice (total 4 min vortex) to fully recover the planktonic cells. The enumeration of bacteria on the LDPE coupon was determined by serial spread-plate dilution on TSAR plates. The bacterial counts were determined after incubation at 37 °C for 48 h and expressed as log cfu·cm^–2^. The antifouling assays were performed in triplicate (*n* = 3).

### 4.11 CV Assay

The developed biofilms post hot water washing treatment were also characterized by CV assays.^26^ In a separate six well plate, the treated coupon was incubated in 2 mL of 0.1% CV aqueous solution in the dark for 20 min at ambient conditions for staining. After the staining, the coupons were washed in 10 mL of PBS buffer twice to remove the unattached CV. The stained coupons were fully dried and subjected to photographing.

### 4.12 Statistical Methods

Data from the experiments were analyzed through the application of a one-way ANOVA for statistical evaluation. Each experimental condition was replicated a minimum of three times (*n* ≥ 3) to ensure reliability. The findings are expressed as the average ± the standard deviation.

## Supporting Information Available

Images of water-LDPE contacting angle after 0–10 min of plasma treatment; pick-up rate of fabricated coating systems on LDPE coupons; and swelling ratio of fabricated coating systems (PDF)
