# Supplementary material for: Protein-Based Rechargeable and Replaceable Antimicrobial and Antifouling Coatings on Hydrophobic Food-Contact Surfaces
Source: ACS Appl Bio Mater. 2024 Feb 28;7(3):1842–51. doi: 10.1021/acsabm.3c01247 (PMC10951945; doi:10.1021/acsabm.3c01247)

Protein-Based Rechargeable and Replaceable Antimicrobial and Anti-fouling Coatings on Hydrophobic Food-Contact Surfaces

Supporting Information

Jiahan Zou [a], Jody Wong [a], Chih-Rong Lee [a], Nitin Nitin [b], Luxin Wang [b], Gang Sun*, [a]

[a] Department of Biological and Agricultural Engineering, University of California, One Shields Avenue, Davis, California, 95616, United States. [b] Department of Food Science and Technology, University of California, One Shields Avenue, Davis, California, 95616, United States

AUTHOR INFORMATION

Corresponding Author

Gang Sun* E-mail: gysun@ucdavis.edu. Tel: (530) 752-0840

Figure S1. Images of water-LDPE contacting angle after 0 – 10 min of plasma treatment.

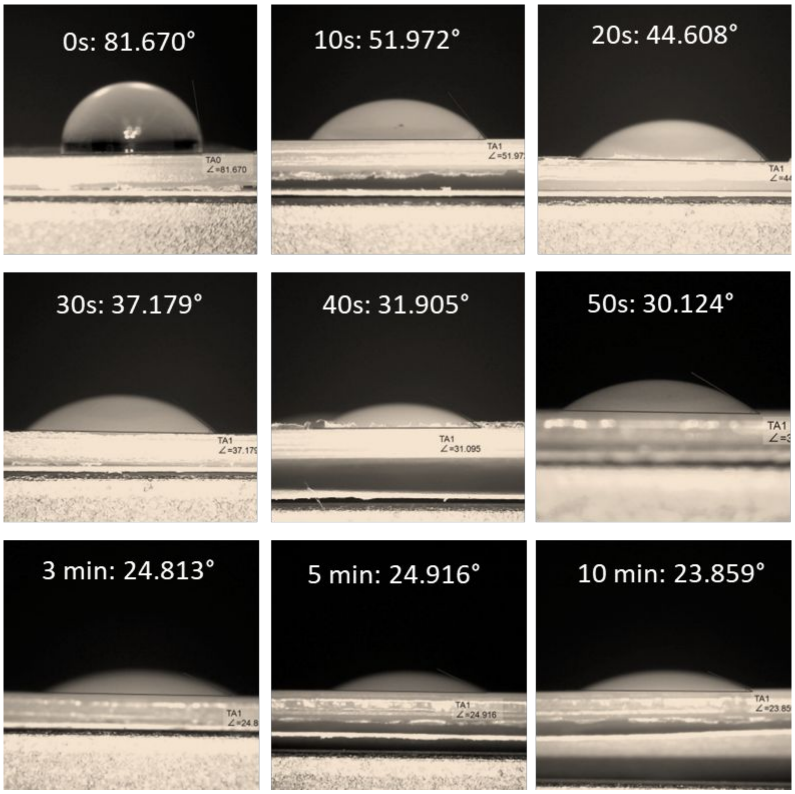

Figure S2. Pick-up rate of Gel (**a**), Gel-based (**b**), and Gel/SPH-based (**c**) coating systems on LDPE coupons. Plotted data are expressed as means ± SD of three replicates.

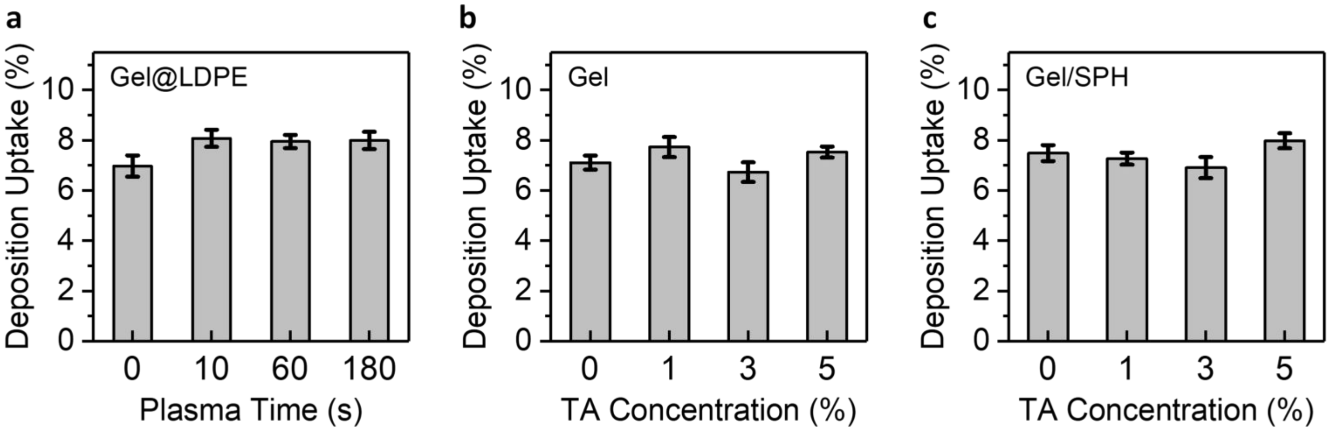

Figure S3. Comparison of swelling ratio of Gel/TA@LDPE and Gel/SPH/TA@LDPE in ambient still water bath.

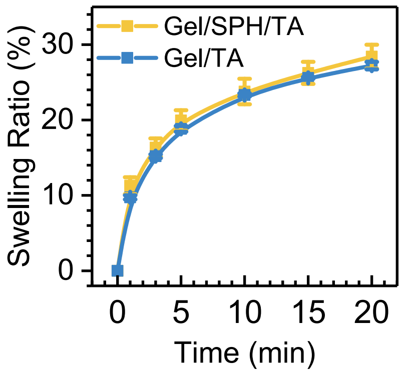

Supplement: Supplementary file 1 — mt3c01247_si_001.pdf [file mt3c01247_si_001.pdf]
